# Insect pests and natural enemies associated with lettuce *Lactuca sativa* L. (Asteraceae) in an aquaponics system

**DOI:** 10.1038/s41598-024-63938-4

**Published:** 2024-06-28

**Authors:** Tamara Machado da Silva, Francisco Jorge Cividanes, Fernando André Salles, Amanda Liz Pacífico Manfrim Perticarrari, Suzan Beatriz Zambon da Cunha, Terezinha Monteiro dos Santos-Cividanes

**Affiliations:** 1 Instituto Biológico, Avenida Bandeirantes, 2419, Ribeirão Preto, São Paulo, CEP 14030-600 Brazil; 2 Instituto de Zootecnia, Avenida Bandeirantes, 2419, Ribeirão Preto, São Paulo, CEP 14030-600 Brazil; 3 Departamento de Engenharia e Ciências Exatas, Universidade Estadual Paulista, Campus de Jaboticabal, Jaboticabal, São Paulo, CEP 14884-900 Brazil; 4 Departamento de Ecologia e Biologia Evolutiva, Universidade Federal de São Carlos, São Carlos, São Paulo, CEP 13565-505 Brazil

**Keywords:** Aquaponics, Vegetable, Thrips, Aphid, Predator, Ladybug, Entomology, Ecology

## Abstract

Although food is produced in aquaponics systems worldwide, no information is available on the occurrence of insect pests and natural enemies in aquaponic lettuce, *Lactuca sativa* L. In this study, a survey was carried out in an aquaponic system combining lettuce with lambari, *Astyanax altiparanae* (Garutti & Briski), aiming to determine the insect pests and natural enemies associated with this system. We also determined the predominant insect species and the effect of meteorological factors on their populations. Insect abundance was estimated by visual sampling during 13 cultivation cycles, totaling 27 sampling dates. The meteorological factors considered were air temperature and relative humidity, and their effects were determined using the Pearson correlation. The thrips *Frankliniella schultzei* (Trybom) and *Caliothrips phaseoli* (Hood) and the aphid *Aphis spiraecola* (Patch) predominated. Ambient temperature and relative humidity were essential factors affecting *C. phaseoli* and *F. schultzei*. The natural enemies found on the lettuce plants were the thrips *Franklinothrips vespiformis* (Crawford) and *Stomatothrips angustipennis* (Hood) and the ladybugs *Cycloneda sanguinea* L., *Eriopis connexa* (Germar), and *Hippodamia convergens* (Guérin-Méneville). These results constitute the first step for a lettuce-integrated pest-management program in aquaponics systems.

## Introduction

The main challenges to world progress and prosperity include eradicating poverty, solutions to water scarcity, promoting sustainable agriculture, and food security^1^. Success in sustainable food production depends on adopting new eco-friendly agricultural technologies such as organic farming and integrated pest management^2^. The aquaponics system is a sustainable production technology based on plants grown in hydroponics integrated with fish farming. This system is based on recycling water and nutrients, with most of the nutrients for plant growth coming from the wastes excreted by fish, without applying fertilizers and pesticides to the plants^3–7^. Aquaponics is an innovative technology “that can change our lives” due to sustainable food production^8^. Simultaneous production of food plants and fish increases the producer's profitability due to less demand for water, reduced soil degradation and environmental contamination, and cost reduction because of less use of chemical pesticides and land area^4,9^.

Aquaponics has been gaining popularity as an option for the sustainable production of organic vegetables and fish, and is used in more than 40 countries, including Brazil. These systems are used in humanitarian activities and as a component of urban and peri-urban agriculture^10,11^. Additionally, aquaponics is used in small-scale food production for subsistence, domestic, and commercial use, including mainly leaf and aromatic crops^3,5,11^. Lettuce, *Lactuca sativa* L., is one of the most important species of green leaf vegetables produced in aquaponics systems^12,13^. Concerning fish farming, tilapia *Oreochromis* spp., catfish (Order Siluriformes), and ornamental fish are the most common types used in aquaponics. The Nile tilapia, *Oreochromis niloticus* (L.), is the most popular fish reared in aquaponics worldwide^12^. As a result, there is abundant information about the economic and productive potential of aquaponic lettuce production combined with tilapia farming^14–19^.

Although world production of food in aquaponics systems has evolved in recent years, there is a lack of information on this technology to control arthropod pests, which makes it essential to develop pest-management strategies to improve these systems^5,6,13^. Published studies on plant protection in aquaponics concerning arthropod pests have reported the incidences of the insect pests *Chironomus* sp. and *Bradysia* sp. in lettuce plants associated with the cultivation of fish in an aquaponic system conducted in a greenhouse^20^. To control the spider mite *Tetranychus urticae* on aquaponic lettuce, Abbey et al.^16^ released predatory mites (*Amblyseius* sp., *Neoseiulus* sp., *Galendromus occidentalis* (Nesbitt), and *Phytoseiulus persimilis* (Athias-Henriot), and used yellow sticky traps for monitoring adults of whitefly (*Trialeuroides vaporariorum* (Westwood)) and thrips (Thysanoptera). da Silva et al.^21^ reported the occurrence of the aphid *Brevicoryne brassicae* L. on cabbage plants and the whitefly *Bemisia tabaci* (Gennadius) on lettuce integrated into a rearing system of the black pacu fish, *Piaractus brachypomus* (Cuvier), determining the efficiency of the entomopathogens *Beauveria bassiana* (Bals.) and *Metarhizium anisopliae* (Metchnikoff) in controlling those pests. Folorunso et al.^6^ reviewed the control measures used in integrated management of pests and diseases in hydroponics cultivation systems and the possibilities of adapting these techniques for aquaponics. A study conducted by^13^ on small producers using aquaponics systems in Latin America and Spain showed that most producers were unaware of or had no experience in operating the systems. Among them, 49% stated that they lacked information about feeding, handling, and fish health management, while 47% mentioned a lack of information about cultivation and protection of plants. According to the authors, the aquaponic systems integrated tilapia farming with cultivation of vegetables such as lettuce, chard (*Beta vulgaris* L.), cabbage (*Brassica oleracea* L. var. *capitata* L.), tomato, cucumber (*Cucumis sativus* L.), basil (*Ocimum basilicum* L.), mint (*Mentha spicata* L.), and coriander (*Coriandrum sativum* L.). Aphids, whiteflies, and thrips were the insect pests found in cultivated vegetables, against which most producers (37%) employed biological control through releases of predators or parasitoids. Despite the problems faced by the producers regarding lack of knowledge on the management of fish and plants in an aquaponics system, most of them used this technology for producing pesticide-free food.

Successful integrated pest-management programs depend primarily on the recognition of insect pests and biological-control agents associated with the crop. In the present study, we conducted a survey in an aquaponic system producing lettuce combined with lambari, *Astyanax altiparanae* (Garutti & Briski), aiming to determine the insect pests and natural enemies present in the system. Lettuce was selected for study because of its high commercial value, high productivity, and short development cycle^3,11^. The lambari, despite its small size (about 10 cm long), is promising for rearing in aquaponics systems, because it has several commercial purposes such as (i) consumption as snacks in bars and restaurants; (ii) live bait for sport or commercial fishing; and (iii) marketing as canned fish, as a sardine-like product^22^.

## Materials and methods

### Study site

The study was carried out in an aquaponic system located in the experimental area of the Escola Estadual Bairro Francisco Castilho (21° 20′ 24″ S and 47° 43′ 46″ W), in Cravinhos municipality, São Paulo state, Brazil.

### Aquaponic system

**Figure 1:**
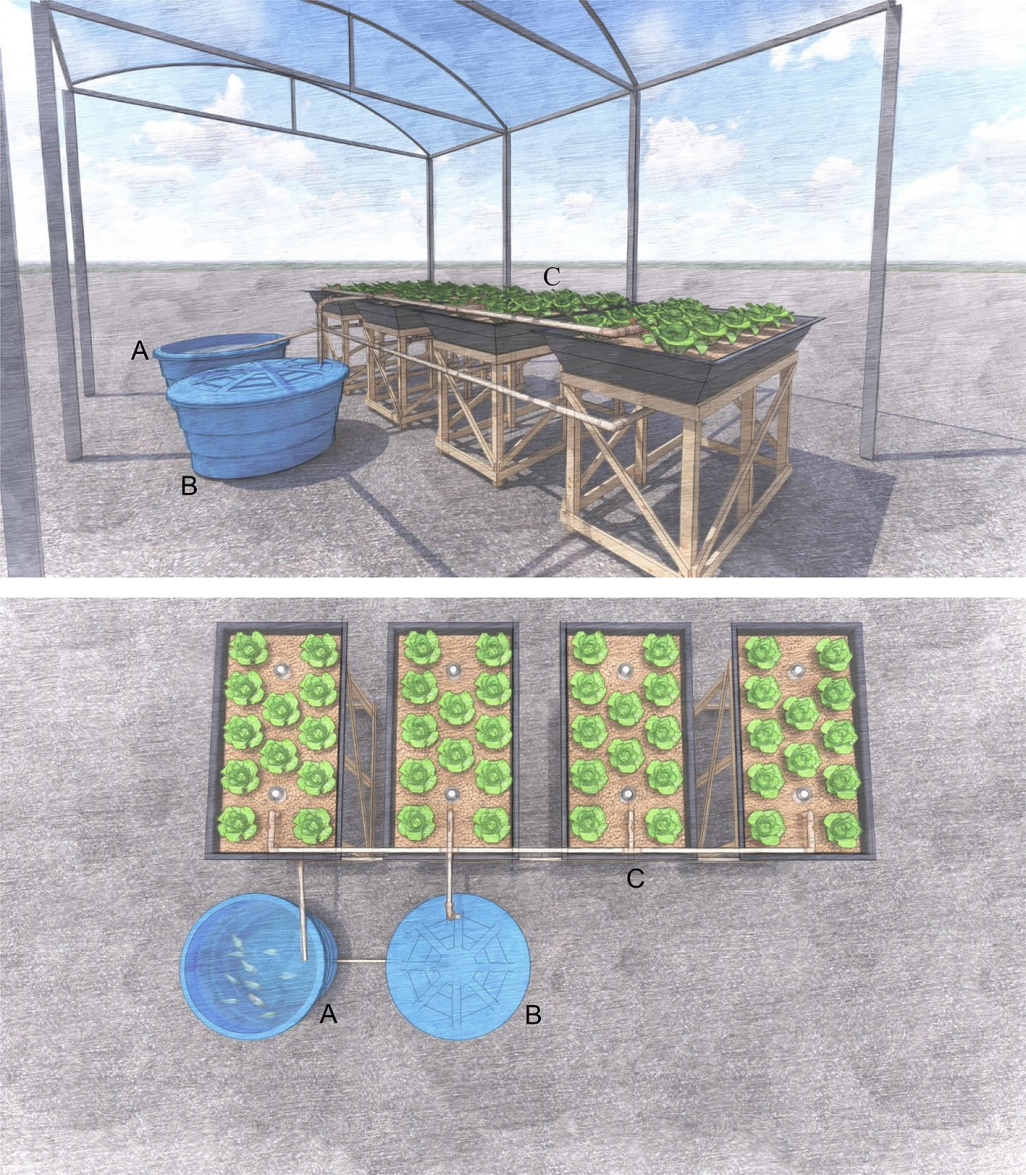
Scheme of aquaponic system with integrated lettuce—lambari culture. Tank A (fish tank), B (pump tank) and C (lettuce). Drawing designed by Isabella Alves Noronha.

The aquaponics system included the following components: four hydroponic beds, each with an area of 2 m^2^ and continually recirculated with a submerged pump. The system had two circular polyethylene tanks (500 L): a fish tank and a sump containing the water pump that distributed water to the entire system (Fig. 1).

Each hydroponic bed had Cinexpan® expanded clay as a substrate, where 15-day-old lettuce seedlings cv. Vanda obtained from a commercial vegetable seedling production nursery were transplanted. The lettuce was harvested after four weeks, according to^3^. Lettuce was cultivated during 13 cycles, which were carried out continuously, from February to September 2021. During April, the system was undergoing maintenance and lettuce cultivation resumed in May. The aquaponics system was maintained weekly, including regular cleaning of the sedimentation filter, water replenishment, and periodic observation of disease and stress symptoms in the fish.

A total of 300 juvenile lambaris (*A. altiparanae*) were kept in a circular polyethylene tank (500 L), with water entering in a tangential flow that helped to remove the fish waste. The water-flow calibration ensured a circulation water-flow rate of 100% per hour were used. Their mean initial weight was 3.0 g (δ = 0.44 g, n = 30) and reached a mean of 36 g (δ = 6.5 g; n = 30) at the end of the experiment. The final estimated total fish biomass (273 individuals) was approximately 9.83 kg, with a final density of 12.3 kg of fish.m^–3^. Fish were fed with extruded feed containing 40% crude protein (CP) and 1.8–2.0 mm in diameter. The feed was offered once or twice a day ad libitum, five days a week. During the experimental period, a total of 12.5 kg of feed was consumed by the fish, reaching a feed efficiency of 70%.

We emphasize that the lambari fish were not the target of evaluations in the present study, i.e., these organisms were kept without being manipulated during the research. The Postgraduate Course Committee “Plant and Animal Health, Food and Environmental Safety in the Agribusiness” at the Biological Institute evaluated and approved the experimental protocols related to lambari fish and lettuce plants. All methods were performed in accordance with the relevant guidelines and regulations of the Biological Institute, São Paulo, SP, Brazil. The lambari fish farming followed the recommendations of the Brazilian Federal Law 11,794 (https://www.planalto.gov.br/ccivil_03/_ato2007-2010/2008/lei/l11794.htm). This study followed Arrive guidelines (https://arriveguidelines.org).

During the experimental period, iron deficiency in the lettuce plants was monitored based on leaf color change, according to^23^. To prevent this, the commercial formulation Rexolin, containing iron chelated with EDDHA 6%, was applied in a concentration of 2 mg L^–1^. Chelated iron was reapplied every 2 months. The ammonia, nitrite, and nitrate concentrations in water were quantified using the API Freshwater Master Test Kit, which uses colorimetric methods to determine the concentration of these chemical compounds. These values served predominantly as a reference point, ensuring that no toxic compounds such as ammonia or nitrite were present. The nitrate concentration was sufficient to promote plant growth. Limnological water parameters (pH, temperature, oxygen saturation, and electrical conductivity; Table 1) were monitored and recorded weekly, in the morning, using an Akso AK88 multiparameter probe. The water was maintained at pH = 7.0 by applying the bases calcium hydroxide and potassium hydroxide alternately at weekly intervals. The fresh weight of 382 plants from 10 harvest periods was determined. The mean fresh weight (including the shoot and roots of each plant) was 170 g (δ = 37.5 g), while the mean fresh weight of the aerial part of the plant was 151 g (δ = 36.3 g).

**Table 1:** Limnological parameters of the lettuce and lambari quaponics cultivation system.

Measure	pH	Temperature (^o^C)	Oxygen saturation (%)	Electric conductivity (µS.cm^-1^)
Mean	6.1	21.9	64.8	232
Standard deviation	0.65	4.3	9.3	79.4
Maximum	7.58	28.4	80.1	381
Minimum	5.14	10.8	35.2	100.4

Cravinhos, São Paulo State, Brazil.

### Sampling insect pests and natural enemies

The abundance of insect pests and natural enemies was determined by visual sampling of the lettuce plants during 13 cultivation cycles, from February to September 2021. During the sampling period, 10 plants per hydroponic bed were randomly selected each week. Insect pests and natural enemies were captured with an entomological aspirator and glass tube (height 8.0 cm, diameter 2.5 cm), respectively. The insect pests and natural enemies were transferred to the Laboratory of Entomology and Biological Control (LECB), Ribeirão Preto, São Paulo, Brazil.

Aphid and thrips specimens were separated using a number zero camel-hair brush and transferred to Eppendorf tubes containing 70% ethanol. The thrips species were identified by Élison Fabrício B. Lima, Laboratory of Bioecology and Systematics of Arthropods, Federal University of Piaui—UFPI, Amilcar Ferreira Sobral Campus—CAFS, Floriano, Piaui. The aphids were identified by Suzan Beatriz Z. da Cunha, Department of Ecology and Evolutionary Biology, Federal University of São Carlos—UFSCar, São Carlos, São Paulo. Exemplars of the insect species are deposited in collections as follows: Coccinellidae: Museum of Entomology of the LECB, Ribeirão Preto, São Paulo; Thysanoptera: Natural History Collection—CHNUFPI, Federal University of Piauí (UFPI—CAFS); and Aphididae: Laboratory of Curatorship of the Aphid Collection—COLEAFIS/DEBE, Federal University of São Carlos (UFSCar).

### Statistical analyses

The faunistic coefficients of dominance, frequency, constancy, and abundance of the insects were determined by faunistic analysis, using the ANAFAU software^24^ The species were classified as predominant when they reached the highest values of the faunistic coefficients, following Silveira^25^.

The effects of the meteorological factors (maximum, minimum, and mean temperatures, maximum and minimum relative humidities, and rainfall) on the abundance of predominant species was examined by the Pearson correlation, using the IBM SPSS Statistics 20 program^26^. The analysis was based on the total number of individuals of the insect species found on each sampling date. Regarding abiotic factors, we used the mean temperature and relative humidity recorded seven days before the sampling date, and the total rainfall in that period. Python IDE (integrated development environment) Jupyter Lab (version 3.7.4 for Windows) software was used to draw the scatter diagrams representing the correlation between thrips occurrence and meteorological factors. The meteorological data were obtained from the Agência Brasileira de Meteorologia Ltda. (Climatempo Agency).

The population fluctuations of the predominant species of insect pests were obtained from graphs relating the number of individuals to mean temperature, relative humidity (maximum and minimum), and rainfall.

### Statement of compliance

The authors declare that the experimental studies involving the collection and use of lettuce plants in this research are in accordance with relevant institutional, national, and international guidelines and regulations.

## Results

### Abundance of insect pests

A total of 4078 individual phytophagous insects belonging to the aphids and thrips groups were captured, during 13 growing cycles of the lettuce aquaponics (Table 2).

**Table 2:** Faunistic coefficients of aphid and thrips species on lettuce plants in an quaponics system, Cravinhos, São Paulo state, Brazil.

Taxon	Number of insects captured	Sampling rate	Dominance	Frequency	Constancy	Abundance
Thysanoptera						
* Caliothrips phaseoli*	1477*	24	SD	SF	W	as
*Echinothrips mexicanus*	1	1	ND	I	Z	d
*Frankliniella insularis*	3	3	ND	F	Y	c
*Frankliniella schultzei*	2552*	25	SD	SF	W	as
*Gynaikothrips* sp*.*	19	3	D	VF	Y	va
*Haplothrips gowdeyi*	2	2	ND	F	Y	c
*Pseudophilothrips* sp.	1	1	ND	I	Z	d
Hemiptera						
*Aphis spiraecola*	12*	9	D	VF	Y	va
*Hyperomyzus lactucae*	2	2	ND	F	Y	c
*Macrosiphum euphorbiae*	1	1	ND	I	Z	d
*Myzus persicae*	3	3	ND	F	Y	c
*Pemphigus bursarius*	5	4	ND	F	Y	c

*Predominant species (indicators). SD, super dominant; D, dominant; and ND, non-dominant. SF, super frequent; VF, very frequent; F, frequent; I, infrequent; W, constant; Y, accessory; Z, accidental; as, super abundant; va, very abundant; c, common; d, dispersed.

The following five species of aphids were identified: *Aphis spiraecola* (Patch)*, Hyperomyzus lactucae* (L.), *Macrosiphum euphorbiae* (Thomas), *Myzus persicae* (Sulzer), and *Pemphigus bursarius* (L.) (Table 2). *Aphis spiraecola* was predominant, found on 9 of the 27 sampling dates, totaling 52% of all aphids collected. The other aphid species were non-dominant, frequent, or infrequent.

The phytophagous thrips collected on the lettuce plants were *Caliothrips phaseoli* (Hood), *Echinothrips mexicanus* (Moulton), *Frankliniella insularis* (Franklin), *Frankliniella schultzei* (Trybom), *Gynaikothrips* sp., *Haplothrips gowdeyi* (Franklin), and *Pseudophilothrips* sp. (Table 2). *Frankliniella schultzei* and *C. phaseoli* were predominant, and the number of individuals observed reached, respectively, 63% and 36% of all thrips collected during the sampling period. *Gynaikothrips* sp. was dominant and very frequent, and *F. insularis*, *E. mexicanus*, *H. gowdeyi*, and *Pseudophilothrips* sp. were non-dominant, frequent, or infrequent, together comprising less than 1% of the total thrips collected (Table 2).

### Population fluctuations and effects of meteorological factors

**Figure 2:**
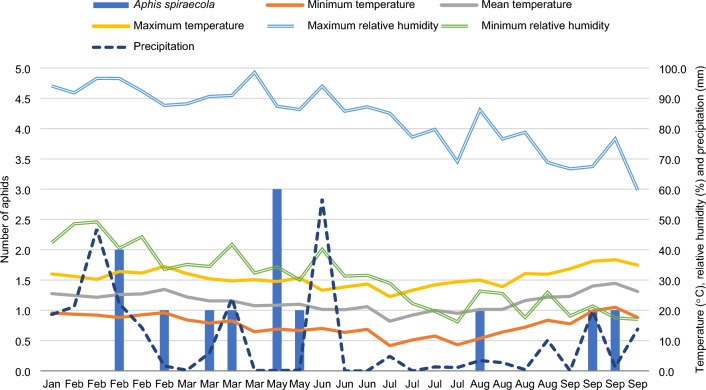
Population fluctuation of *Aphis spiraecola* in lettuce grown in an aquaponic system. Cravinhos, São Paulo state, Brazil.

Except for June and July, *A. spiraecola* occurred in most of the samples taken from February to September (Fig. 2). During June and July, the mean ambient temperature was 19.4 °C, relative humidity 54.9%, and total rainfall 64.0 mm. In the remaining months these factors reached 24.1 °C, 57.6%, and 188.0 mm, respectively. The correlation between *A. spiraecola* and the meteorological factors was not significant (Table 3).

**Table 3:** Correlation coefficients (r) between the density of aphids and thrips and abiotic factors in lettuce in an aquaponic system.

Aphid and thrips species	Temperature			Relative humidity		Precipitation
	Min	Me	Max	Min	Max	
*Aphis spiraecola*	0.16^ns^	0.21^ns^	0.22^ns^	0.11^ns^	0.17^ns^	− 0.06^ns^
*Caliothrips phaseoli*	− 0.52**	− 0.51**	− 0.45*	− 0.34^ns^	− 0.21^ns^	− 0.30^ns^
*Frankliniella schultzei*	0.23^ns^	0.35^ns^	0.46*	− 0.51**	− 0.60**	− 0.13^ns^

Cravinhos, São Paulo state, Brazil.

Min: minimum; Me: mean; and Max: maximum. ns: non-significant; ** and *: significant at 1% and 5%, respectively.

**Figure 3:**
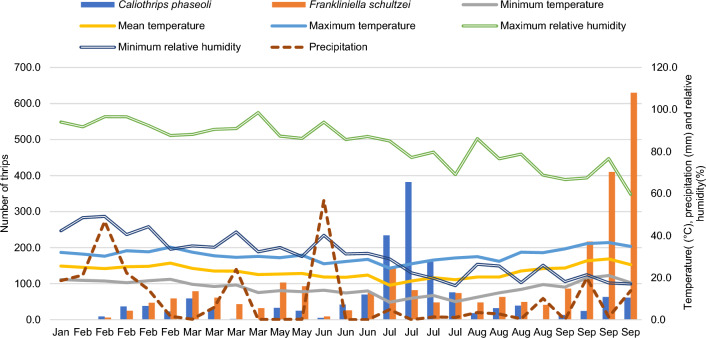
Population fluctuations of *Caliothrips phaseoli* and *Frankliniella schultzei* in lettuce grown in an aquaponic system. Cravinhos, São Paulo state, Brazil.

The population fluctuations of *C. phaseoli* and *F. schultzei* were similar throughout the study period. These thrips were usually abundant from February to May, June to July, and September, coinciding with a period of low rainfall (Fig. 3). The highest population peak of *C. phaseoli* and *F. schultzei* occurred, respectively, in July and September.

**Figure 4:**
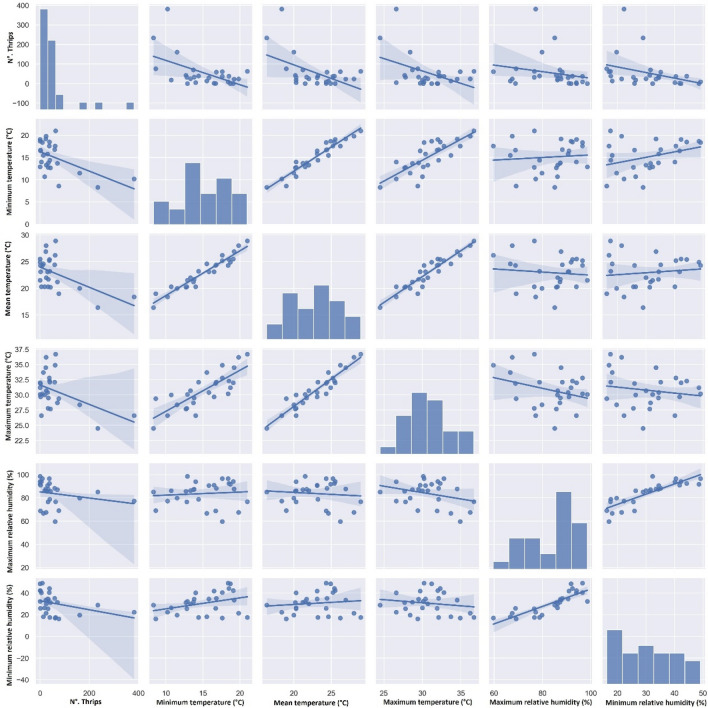
Scatter diagrams representing the correlation between *Caliothrips phaseoli* occurrence and meteorological factors in a lettuce aquaponic system. Cravinhos, São Paulo state, Brazil. Correlation graph among variables N°. Thrips, Minimum temperature (°C), Mean temperature (°C), Maximum temperature (°C), Maximum relative humidity (%), and Minimum relative humidity (%), implemented in Python using the seaborn package^27,28^.

**Figure 5:**
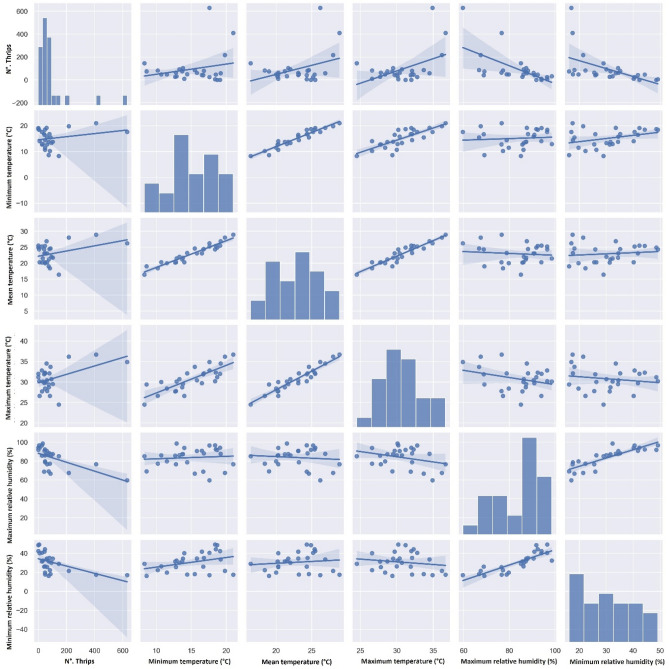
Scatter diagrams representing the correlation between *Frankliniella schultzei* occurrence and meteorological factors in lettuce aquaponic system. Cravinhos, São Paulo State, Brazil.

The correlation analysis showed a significant negative relationship between the maximum (r = − 0.45; *p* < 0.05), minimum (r = − 0.52; *p* < 0.01), and medium (r = − 0.51; *p* < 0.01) temperatures and the density of *C. phaseoli* (Table 3; Fig. 4), while *F. schultzei* showed a significant negative correlation with the maximum (r = − 0.60; *p* < 0.01) and minimum (r = − 0.51; *p* < 0.01) relative humidity and also a significant positive correlation with the maximum temperature (r = 0.46; *p* < 0.05), (Table 3; Fig. 5). On the other hand, *C. phaseoli* showed no significant correlation with the maximum (r = − 0.21; *p* > 0.05) or minimum (r = − 0.34; *p* > 0.05) relative humidity. A similar correlation occurred in *F. schultzei* with mean (r = 0.35; *p* > 0.05) and minimum (r = 0.23; *p* > 0.05) temperatures. Neither thrips species showed a significant correlation with rainfall (r = − 0.30; *p* > 0.05 for *C. phaseoli* and r = − 0.13; *p* > 0.05 for *F. schultzei*).

### Ocurrence of natural enemies

Six species of predatory insects representing two orders were captured and identified as follows: (i) ladybugs (Coleoptera: Coccinellidae), *Eriopis* connexa (Germar) with three individuals captured; *Cycloneda sanguinea* L. (one individual); and *Hippodamia convergens* (Guérin-Méneville) (one individual); and (ii) thrips (Thysanoptera: Aeolothripidae), *Franklinothrips vespiformis* (Crawford) (one individual), and *Stomatothrips angustipennis* (Hood) (one individual).

## Discussion

Aphids are economically important pests of lettuce. Besides the damage and the loss of lettuce quality from their feeding, aphids are vectors of viruses that cause lettuce diseases^29,30^. Although in this study, five species of aphids were found associated with lettuce, only the green citrus aphid, *A. spiraecola*, stood out as very abundant and frequent. However, this species was not found in June and July, the austral winter. *Aphis spiraecola* is one of the most abundant species in lettuce crops^31^ but has not been reported to damage lettuce. *Aphis spiraecola* is polyphagous, occurs worldwide, and is a recognized pest of citrus, apple trees, and ornamental plants, and a vector of several species of phytoviruses^32,33^.

The green peach aphid, *M. persicae*, is an important pest worldwide. This aphid causes up to 50% loss of yield in vegetable crops, due to phytovirus transmission^34^. In Brazil, *M. persicae* comprised 38% of individuals of three aphid species found in hydroponic lettuce^35^. The potato aphid, *M. euphorbiae*, has a wide host range and is also an important pest worldwide, due to its transmission of several phytoviruses^36^. In Brazil, *M. euphorbiae* is one of the main pests of lettuce in protected cultivation, and a vector of the LMV virus^37^. This aphid has been reported in hydroponic lettuce in São Paulo state^35^ and in lettuce fields in Paraná state^34^. Additionally, *H. lactucae* and *P. bursarius* are common in many countries^38–40^ and can be vectors of plant viruses in lettuce^41–43^.

In the present study, the small number of aphids found on the lettuce plants may be related probably to the lettuce stiffness leaf. This lettuce cultivar in aquaponic system showed leaves with a stiffer texture than when it was grown in soil. Ibrahim and Zuki^44^ found that the Grand Rapid lettuce cultivar had a significantly higher percentage of fiber in an aquaponic system compared to lettuce grown in hydroponics and soil. According to^45^ the dietary fiber found in vegetables, cereals, and fruits is composed of polysaccharides, cellulose, hemicellulose, lignin, pectins, gums, and oligosaccharides, which are recognized as resistance factors of plants to insect pests in cotton, potatoes, and forage plants^46–48;^ lignin is a particular component of resistance to aphids.

Studies on varietal resistance to pests are essential to the genetic improvement of lettuce^29^. However, the possible differences in resistance to lettuce pests in aquaponic, hydroponic, and soil systems remain to be clarified. We suggest investigating the relationship of dietary-fiber components of lettuce cultivars to the resistance of this vegetable against aphids and thrips, with a focus on developing strategies to manage pests in aquaponic systems.

Although the diversity of pests and natural enemies in lettuce grown in field and hydroponic conditions has been widely studied^34,35,49–52^, we are unaware of published studies on the occurrence of these organisms in aquaponic lettuce. This is an essential step in the development of an innovative system such as aquaponic lettuce.

In the present study, *F. schultzei* and *C. phaseoli* constituted 99% of the captured specimens among the five thrips species found in lettuce. *Frankliniella schultzei* and *C. phaseoli* are considered the most abundant and harmful in lettuce grown in the field and hydroponically^34,52^. The former is a vector of tospoviruses (genus *Tospovirus*, family Bunyaviridae), an important group of viruses affecting many plant species, including lettuce. Furthermore, when feeding on lettuce leaves, *F. schultzei* causes chlorotic spots that reduce the commercial quality^34,42,53,54^.

The low incidence of *Gynaikothrips* sp., *F. insularis*, *H. gowdeyi*, *E. mexicanus*, and *Pseudophilothrips* sp. may indicate that these thrips occurred incidentally in the lettuce plants. Only *F. insularis* has been reported to damage lettuce. *Frankliniella insularis* has a wide distribution in the Americas and is reported to cause considerable damage to lettuce on the islands of Guadeloupe and Martinique, and also as a secondary pest of citrus in Brazil^55,56^. *Haplothrips gowdeyi* is widely distributed in tropical and subtropical countries, occurring in flowers of a wide variety of plants^56^. *Echinothrips mexicanus* occurs in lettuce, and cassava^52,57^. No published studies have reported *Pseudophilothrips* sp. on lettuce. However, the genus *Pseudophilothrips* is a pest of guava, and its damage compromises the fruits' aesthetic value and increases the plant's susceptibility to fungal infestation^58^. The genus *Gynaikothrips* includes 16 species associated with *Ficus* (Moraceae) that cause considerable damage to plant leaves^59^.

Environmental temperature was the most important abiotic factor affecting the incidence of *C. phaseoli* in lettuce, while temperature and relative humidity were essential factors affecting *F. schultzei*. According to^60^, temperature and rainfall are meteorological factors responsible for up to 65% of the numerical variation of thrips in an agroecosystem. In the present study, the abundance of *C. phaseoli* decreased in periods with mean temperatures above 30 °C during summer and late winter, while its maximum population peak occurred in winter, with a mean temperature of 26.6 °C. The two highest population peaks of *F. schultzei* coincided with rising temperatures in early spring, with the maximum peak occurring at the mean temperature of 26.2 °C. Morsello et al.^60^ also found population growth of *Frankliniella fusca* (Hings) with rising temperatures in spring.

The lowest population densities of *C. phaseoli* and *F. schultzei* occurred in periods of more intense rainfall. Rainfall contributes significantly to the mortality of insect pests, affecting them both directly (washing and drowning individuals) and indirectly by favoring the occurrence of entomopathogenic fungi^61–64^. The aquaponic system used here was in a semi-field environment where the lettuce plants were not directly exposed to rain. As mentioned above, meteorological factors such as temperature, and especially rainfall, can have a significant impact on thrips abundance^60,65–67^. The significant negative correlation between relative humidity and *F. schultzei* obtained here may indicate an indirect influence of rain that limited the flight capacity of thrips, reducing dispersal and, consequently, colonization of lettuce plants in periods of rainfall.

Barrière et al.^29^ highlighted the importance of favorable climatic conditions for the incidence of pests in lettuce crops. In view of the present results, it is essential to conduct studies on the influence of meteorological factors such as temperature, relative humidity, and rainfall on *C. phaseoli* and *F. schultzei*. Understanding the effects of abiotic factors on a thrips population is necessary for an effective and sustainable control strategy, aiming to minimize thrips damage and increase lettuce quality and productivity.

Among the species of predatory thrips collected on lettuce, *F. vespiformis* feeds on small arthropods, including mites, whiteflies, and thrips. The species is an important control agent for thrips of the genus *Scirtothrips* that attack avocado trees^56^. *Stomatothrips angustipennis* occurs only in Brazil, but there is no information about its economic importance in agriculture. However, the species is a predator of mites and other thrips species^56^.

Ladybugs are polyphagous, feeding on aphids, mealybugs, whiteflies, and mites^68^. *Cycloneda sanguinea* and *H. convergens* are aphidophagous species^69–72^, while *E. connexa* feeds on aphids, mites, and lepidopteran eggs^73,74^. *Hippodamia convergens* is a potential control agent of the lettuce pests *M. persicae* and *Thrips tabaci* (Lind) as noted by^75^.

The low density of ladybugs found here may be related to the low occurrence of aphids on the plants, since aphids are essential food for most species of ladybugs^76^. The lack of vegetation near the aquaponic system may also have contributed to the low incidence of these natural enemies. A plant shortage around aquaponic lettuce can be modified by providing sources of food and shelter to maintain and increase populations of natural enemies^77^. According to^78^, diversification of plant species can increase the abundance, diversity, and dispersal of alternative prey, enhancing generalist predator populations. Sengonca et al.^79^ reported that plants of *Artemisia vulgaris* L. (Asteraceae), *Tanacetum vulgare* L. (Asteraceae), and *Urtica dioica* L. (Urticaceae) adjacent to a lettuce crop increased the number of larvae and adults of *Coccinella septempunctata* L., *Adalia bipunctata* L., and *Propylea quatuordecimpunctata* L., which reduced the populations of *M. persicae, Nasonovia ribisnigri* Mosley, and *M. euphorbiae*.

This study indicated that thrips and aphids are significant pests of aquaponic lettuce, and natural enemies were represented by ladybugs and predatory thrips, occurring in low density. On the other hand, we are aware of no published studies on control of pests in aquaponic lettuce^6^, and conservation biological control is appropriate for aquaponics technology. This situation opens avenues of opportunity for developing pest-management strategies such as habitat manipulation, which has wide applications in agriculture^80^. For example, to increase ladybug populations in aquaponic lettuce, we can recommend establishment of other plant species that do not host lettuce pests and have periods of intense flowering in the vicinity of the system.

## Data Availability

The datasets obtained and analyzed during the current study are available from the corresponding author on reasonable request.
